# Editorial: Ocular infection of herpes: Immunology, pathogenesis, and interventions

**DOI:** 10.3389/fmicb.2022.986859

**Published:** 2022-07-26

**Authors:** Tejabhiram Yadavalli, Chandrashekhar Patil, Dinesh Jaishankar, Rahul Suryawanshi

**Affiliations:** ^1^Department of Ophthalmology and Visual Science, University of Illinois at Chicago, Chicago, IL, United States; ^2^Division of Surgery, Department of Pediatrics, Comprehensive Transplant Center, Feinberg School of Medicine, Northwestern University, Chicago, IL, United States; ^3^Gladstone Institute of Virology, Gladstone Institute, San Francisco, CA, United States

**Keywords:** herpes, pathogenesis, vaccines, eye, cornea, trigeminal ganglia

Ocular infection of herpes: Immunology, pathogenesis, and interventions

Ocular viral infections, caused by the human herpesvirus family, manifests as corneal and stromal keratitis (Lobo et al., 2019; Koganti et al., 2021), conjunctivitis (Shaikh and Ta, 2002), uveitis (Chee et al., 2008), or retinitis (Munro et al., 2019), potentially resulting in vision loss if not diagnosed and treated in a timely manner. While viral replication on the corneal surface occurs for a limited period, host response to infection in the form of inflammation results in the pathologies mentioned above. In this regard, it is of utmost importance to uncover aspects of the host-virus interactions that occur on the ocular surface to mitigate damage to the eye. Furthermore, a better understanding of immunology and pathogenesis of ocular virus infections can uncover novel drug targets to develop interventions and therapeutics to help millions of people around the world who suffer from ocular herpes infection.

Given the complexity of the ocular tissue and its immune privilege status (Liu and Li, 2021), it is crucial to understand the mechanisms of viral entry and replication in corneal tissues and host factors that either aid or deter viral infection to develop novel therapeutics and vaccines (Koganti et al., 2019). Over the past decades, significant progress has been made in all these aspects to tremendously improve our understanding of the pathophysiology of viral infections. This Research Topic is a collection of articles that further improve our understanding. Specifically, the themes observed in this Research Topic are focused on viral entry mechanisms, host factors influencing viral entry and replication, and recent advances in vaccines against ocular herpesviruses.

Wang et al. identified nectin-1 and non-muscle myosin heavy chain-IIB (NMHC-IIB) as mediators of herpes simplex virus-1 (HSV-1) entry into corneal nerves. While several host receptors that mediate HSV entry have been identified, receptor(s) that mediate entry of HSV-1 specifically into corneal nerves have remained elusive. In their original research article, through *in vitro* siRNA knockdown and *in vivo* antibody blocking, they show that nectin-1 and NMHC-IIB are specifically important for the virus to enter primary trigeminal ganglion neurons nerves.

Chang et al. showcase the importance of filopodia induction during human cytomegalovirus (CMV) entry into human iris stromal cells. Filopodial projections are exploited by many viruses to enter and spread within the infected tissue. This interesting study, through confocal imaging, reveals many CMV particles attached to the filopodial structures prior to cell entry. Furthermore, depolymerization of actin filaments in the target cells caused a significant reduction in CMV entry into the host cell. Through genetic upregulation of viral gB protein in these cells, the authors show a significant induction in filopodial structures and a complete loss when the cells were pre-treated with anti-3-OS HS (G2) peptide and/or heparinase-I. These exciting results point to the possibility of actin cytoskeleton modulation through viral gB and host 3-OS HS receptor. Along similar lines, Yang et al., in their review article, discuss the importance of the conserved herpesvirus tegument protein UL11 and its binding partners. This review discusses the roles of UL11 in viral assembly, primary and secondary envelopment, and cell-to-cell transmission to obtain a better understanding of the UL11 protein in the life cycle of herpesviruses and to serve as a reference for studying other viruses.

The article by Battaglia et al. demonstrate the role of host factors in influencing viral pathogenies. The authors demonstrate that disease severity is directly proportional to HSV-1-induced serotonin levels in the eye. They found that HSV-1 profoundly altered the gene expression patterns of amino acid biosynthetic pathway molecules including key tryptophan metabolism genes. These genes were identified as part of an RT PCR Amino Acid Metabolism Array. Further investigation revealed that Tryptophan hydroxylase 2 (TPH2), neuronal-specific rate-limiting enzyme for serotonin, was most significantly upregulated in this data set. Given the role of serotonin in HSV replication and associated pathogenesis is largely explored, the authors in this study have used *in vitro* and *in vivo* models to study how HSV-1 modulates serotonin levels and how it affects the overall pathology of the disease. Their results indicate that HSV-1 promotes serotonin synthesis, which in turn facilitates viral replication and enhancement of pro-inflammatory effects resulting in worsening of ocular disease. A more comprehensive review on the topic of host factors that exacerbate or mitigate viral pathogenesis was provided by Shukla and Valyi-Nagy. In their review, the authors summarize the latest advancements in knowledge regarding host molecules that promote pathophysiological aspects of ocular herpes. The article not only provides a basic introduction to viral structure and lifecycle but also delineates the latest developments in experimental drugs against HSV-1. In a review article by Guo, the author summarizes the importance of cell-death-dependent host factors in protecting against ocular HSV infections. This article sheds light on how HSV-1 modulates host apoptotic, necrotic, necroptotic, and pyroptotic pathways to evade detection and replicate.

Krishnan and Stuart in their review article discuss the most recent advancements in the development of anti-HSV vaccines. It is pertinent to note that there are currently no approved therapeutic or prophylactic vaccines against HSV. However, both preventative and therapeutic vaccines are being developed. This review article highlights the current guidelines for the treatment of HSV infections and the novel vaccine candidates in development.

In conclusion, the collection of studies in this Research Topic further advances our understanding of herpes virus entry and replication and the influence of host factors on viral infection. It will be important to assess how some of these findings can be translated to develop novel interventions either in the form of therapeutics or vaccines.

## Author contributions

All authors listed have made a substantial, direct, and intellectual contribution to the work and approved it for publication.

## Conflict of interest

The authors declare that the research was conducted in the absence of any commercial or financial relationships that could be construed as a potential conflict of interest.

## Publisher's note

All claims expressed in this article are solely those of the authors and do not necessarily represent those of their affiliated organizations, or those of the publisher, the editors and the reviewers. Any product that may be evaluated in this article, or claim that may be made by its manufacturer, is not guaranteed or endorsed by the publisher.
